# Acquired haemophilia A associated with autoimmune thyroiditis: a case report

**DOI:** 10.1186/1752-1947-8-469

**Published:** 2014-12-29

**Authors:** Upul GP Pathirana, Nirmali Gunawardena, Hiranya Abeysinghe, Hannah Charlotte Copley, MG Dhammika Somarathne

**Affiliations:** 16 General Medical Unit, Teaching Hospital, Kandy, Sri Lanka; 17 Department of Surgery, Addenbrooke’s Hospital, University of Cambridge, Cambridge, UK

**Keywords:** Acquired haemophilia, Autoimmune thyroiditis, Bypassing agents, Factor VIII inhibitors, Haemophilia A, Immunosuppression

## Abstract

**Introduction:**

Acquired haemophilia A is a rare life- and limb-threatening bleeding disorder if left untreated. Autoimmune thyroiditis is an autoimmune disorder that can be rarely associated with acquired haemophilia. Here we report a case of a 60-year-old woman presenting with cutaneous and muscle haematomas secondary to acquired haemophilia A in association with autoimmune thyroiditis, who was successfully treated with recombinant activated factor VII and immunosuppression.

**Case presentation:**

A 60-year-old Sri Lankan woman with a background of longstanding hypothyroidism, diabetes mellitus, hypertension, hyperlipidaemia and bronchial asthma developed spontaneous cutaneous purpura and a limb-threatening intramuscular haematoma. Initial coagulation screening revealed prolonged activated partial thromboplastin time of 66.4 seconds (normal range 26 to -36 seconds) and time-dependent inhibitors against factor VIII. She had positive antinuclear antibody and antithyroid peroxidase (microsomal) antibody titre of over 1/80 and 1000IU/mL respectively. The diagnosis was therefore made of acquired haemophilia A in association with autoimmune thyroiditis. Acute limb-threatening bleeding was managed with recombinant activated factor VII (NovoSeven^®^). Immunosuppressive treatment consisting of oral prednisone 60mg/day and cyclophosphamide 100mg/day was administered in order to remove the factor VIII inhibitor. This treatment led to normalisation of her haemostatic parameters. This case illustrates a very rare association of acquired haemophilia and autoimmune thyroiditis as well as the importance of considering acquired haemophilia as a differential diagnosis of spontaneous bleeding.

**Conclusions:**

Acquired haemophilia should be considered in the differential diagnosis of unexplained bleeding in adults. Treatment of the acute coagulopathy with recombinant activated factor VII and immunosuppressive therapy was successful in this case.

## Introduction

Acquired haemophilia A is an autoimmune disease caused by inhibitory antibodies to factor VIII. It often presents with severe and life-threatening bleeding, requiring a rapid intervention of bleeding control and immunosuppression [1]. The diagnosis should be considered in adult patients presenting with spontaneous bleeding along with unexplained, isolated and prolonged activated partial thromboplastin time (aPTT). Moreover, several groups of medical conditions are associated and patients should therefore be investigated for autoimmune diseases, malignancy, pregnancy and dermatological disorders [1]. Here we report a rare case of acquired haemophilia A in association with autoimmune thyroiditis that was successfully treated with immunosuppressive therapy.

## Case presentation

A 60-year-old Sri Lankan woman with longstanding hypothyroidism, diabetes mellitus, hypertension, hyperlipidaemia and bronchial asthma presented to a general medical ward with a recent history of a large, spontaneous, painless bruise over her right thigh. Medication included low dose aspirin 75mg daily. There was no family history of bleeding disorders and she was haemodynamically stable. An ultrasound scan excluded coexisting deep soft tissue haematomas and a full blood count demonstrated a white blood cell count of 11.2×10^9^/L with normal differentials, haemoglobin level of 12.3g/dL and a platelet count of 258×10^9^/L. Coagulation screening revealed an aPTT of 66.4 seconds with normal bleeding, prothrombin and thrombin time, results that were confirmed over repeated assays. The results of her blood films, urea, electrolytes, creatinine and liver function tests were all normal.

Further investigation in our haematology unit demonstrated the presence of a time-dependent inhibitor of coagulation via prolonged aPTT and a mixing study that did not correct with the addition of normal plasma and incubation for 2 hours (aPTT was 52 seconds when the mixing test was performed, with a ratio of her plasma to normal plasma of 50:50). A mixing study of incubated and fresh mixed plasma did not demonstrate a temperature-dependent inhibitor of coagulation (aPTT was 27 seconds with a ratio of her plasma to normal plasma of 50:50). Clotting factor VIII assay and inhibitor titres were not possible due to a lack of facilities. An indirect assay of deficient factor was carried out by adding factor VIII or IX deficient plasma to her plasma. The aPTT was corrected by adding factor IX deficient plasma, but not by factor VIII deficient plasma, thus suggesting factor VIII deficiency. Plasma fibrinogen was 260mg/dL (150 to 250) and platelet aggregation studies were compatible with the expected aspirin-induced changes. This therefore suggested a diagnosis of acquired haemophilia A. Investigation for associated conditions revealed positive antinuclear antibody (ANA) and antithyroid peroxidase (anti-TPO; microsomal) antibody titre of over 1/80 and 1000IU/L respectively. Her thyroid-stimulating hormone (TSH) level was 4mU/L (normal range 0.3 to 4.2mU/L) during the present admission. A previous hyperthyroid state with TSH of <0.01mU/L and free thyroxine (T_4_) of 2.87ng/dL had led to the present hypothyroidism with a corrective thyroxine replacement therapy of 100μg daily. The present state of hypothyroidism with a high titre of anti-TPO antibody was suggestive of autoimmune thyroiditis. Anti-double-stranded DNA was negative. Tests for lupus anticoagulant and anti-cardiolipin antibody were negative. These tests were carried out due to the isolated prolonged aPTT and the positivity of ANA respectively.

Acquired haemophilia A in association with autoimmune thyroiditis was therefore diagnosed. It was promptly treated with a combination of oral prednisolone 60mg daily and alendronate, calcium and vitamin D therapy for bone protection.

Four weeks later she presented with painful swelling of her right calf muscle. There were no features of compartment syndrome and the diagnosis of deep muscle haematoma was confirmed by ultrasound examination. The aPTT was 96 seconds on admission. Acute limb-threatening bleeding was successfully managed with recombinant activated factor VII (NovoSeven^®^). Cyclophosphamide 100mg daily was added to the immunosuppressive regime to induce remission. Eight weeks later, aPTT returned to within the normal range and there were no further haemorrhagic manifestations.

## Discussion

Acquired haemophilia A is primarily seen in the elderly with an incidence of 1.3 to 1.4 per million/year in the United Kingdom [2]. Although uncommon, this condition is associated with a high rate of morbidity and mortality as severe bleeding occurs in up to 90% of affected patients [3]. Acquired haemophilia A is caused by polyclonal autoantibodies to factor VIII, usually immunoglobulin G [4]. Patients typically present with spontaneous subcutaneous bruising, mucosal bleeding (gastrointestinal, lung or urogenital), deep soft tissue bleeding (intracranial, muscle and retroperitoneal haematomas) or iatrogenic bleeding following invasive procedures [1].

The diagnosis of acquired haemophilia A should be considered if cutaneous or soft tissue bleeding is present in elderly patients with no previous history of bleeding or if there is unexplained isolated aPTT prolongation [5]. Acquired haemophilia A is secondary to anti-factor VIII antibodies; these are time dependent and temperature dependent and investigations show an aPTT that does not correct with normal plasma addition, even after incubation. The diagnosis is confirmed by the finding of a low factor VIII and a raised inhibitor titre on Bethesda assay [1]. Our patient, who did not have a past history of bleeding, presented with cutaneous bleeding and was found to have isolated prolonged aPTT. The differential diagnosis for the latter finding includes heparin contamination, clotting factor deficiency, lupus anticoagulant, acquired haemophilia A and von Willebrand disease (vWD) [1]. Our patient was not heparinised and contamination was excluded by proper sampling and repeated assays. Paradoxically, the antiphospholipid syndrome is usually associated with a tendency to thrombosis rather than bleeding; the prolonged aPTT is an artefact of the antiphospholipid phenomenon [6] and this diagnosis was excluded by dint of negative lupus anticoagulant screening. Although her normal bleeding time would not suggest vWD, platelet aggregation studies were nevertheless carried out and simply showed results compatible with aspirin-induced changes. Mixing her plasma with pooled normal plasma (immediate and incubated) can differentiate three other diagnoses of clotting factor deficiency, acquired haemophilia A and vWD. The presence of time-dependent clotting factor inhibitors excluded the clotting factor deficiency and vWD. We concluded that the inhibitors are against factor VIII by doing further mixing studies with factor VIII and IX deficient plasma as the immunologic and functional assays should give equivalent results when a factor deficiency is present. In the literature, the most common autoantibodies that affect clotting factor activity and lead to a bleeding disorder are, as in this case, directed against and interfering with the activity of factor VIII [7].

Clinical associations of acquired haemophilia include autoimmune diseases (such as rheumatoid arthritis, polymyalgia rheumatic and systemic lupus erythematosus), malignancy (occasionally occult), pregnancy and dermatological disorders, such as pemphigoid [1]. Autoimmune thyroiditis is also known to be an autoimmune disorder which is associated with acquired haemophilia A [7]. A search on PubMed did not reveal any similar case reports. Our patient initially had a thyrotoxic phase followed by euthyroid phase and is now hypothyroid. This clinical picture along with a highly positive titre of anti-TPO (microsomal) antibody is compatible with the diagnosis of autoimmune thyroiditis.

The main principles of treatment for acquired haemophilia A are to control bleeding, to eradicate the inhibitor, to treat underlying disorders and to avoid high risk of trauma including iatrogenic procedures [1].

Bypassing agents are currently the most commonly used first-line treatment and both recombinant activated factor VII and factor VIII inhibitor bypassing activity (FEIBA; the only currently available activated prothrombin complex concentrate) have been shown to be effective in acquired haemophilia A [8]. The concentrates of human factor VIII are less effective in the presence of high titre of factor VIII inhibitors. Here, recombinant activated factor VII was used to control the acute limb-threatening bleeding. In addition, immunosuppressive therapy should be commenced promptly after the diagnosis has been made [5] in order to eradicate the antibody inhibitor. The main options for immunosuppression are steroids, cytotoxics (cyclophosphamide, azathioprine or combination therapy), rituximab, ciclosporin A, plasmapheresis or immunoabsorption and factor VIII immune tolerance. We initially prescribed 60mg of prednisolone per day with the addition of cyclophosphamide after a further bleeding event and no biochemical improvement. The patient made a good recovery and she was free of haemorrhagic manifestations with a normalisation of the aPTT after 8 weeks of starting combined immunosuppressive therapy. Furthermore, she underwent successful cataract surgery 6 months later without any complications.

## Conclusions

Acquired haemophilia A is exceedingly rare and there is therefore a lack of therapeutic guidelines. Although several therapeutic options are available, outcome data are lacking. However, early recognition together with the initiation of inhibitor eradication therapy may reduce the morbidity and mortality associated with this condition.

## Consent

Written informed consent was obtained from the patient for publication of this case report and any accompanying images. A copy of the written consent is available for review by the Editor-in-Chief of this journal.

## Authors’ information

GPUP MBBS Registrar in Medicine

JNG MBBS, MD Consultant physician

AHMHIA MBBS, MD Resident physician

HCC MB BChir BA Honorary Clinical Fellow, Junior Doctor

MGDS MBBS, MD Senior Registrar in Medicine

## Abbreviations

ANA: Antinuclear antibody
anti-TPO: Antithyroid peroxidase
aPTT: Activated partial thromboplastin time
TSH: Thyroid-stimulating hormone
vWD: Von Willebrand disease.
